# Photopatternable Magnetic Hollowbots by Nd-Fe-B Nanocomposite for Potential Targeted Drug Delivery Applications

**DOI:** 10.3390/mi9040182

**Published:** 2018-04-13

**Authors:** Hui Li, Jing Chen, Jinjie Zhang, Jingyong Zhang, Guoru Zhao, Lei Wang

**Affiliations:** Institute of Biomedical & Health Engineering, Shenzhen Institutes of Advanced Technology, Chinese Academy of Sciences, Shenzhen 518055, China; jing.chen@siat.ac.cn (J.C.); jj.zhang@siat.ac.cn (J.Z.); jy.zhang@siat.ac.cn (J.Z.); gr.zhao@siat.ac.cn (G.Z.)

**Keywords:** microfabrication, photopatternable, magnetic composite, hollowbots, drug delivery

## Abstract

In contrast to traditional drug administration, targeted drug delivery can prolong, localize, target and have a protected drug interaction with the diseased tissue. Drug delivery carriers, such as polymeric micelles, liposomes, dendrimers, nanotubes, and so on, are hard to scale-up, costly, and have short shelf life. Here we show the novel fabrication and characterization of photopatternable magnetic hollow microrobots that can potentially be utilized in microfluidics and drug delivery applications. These magnetic hollowbots can be fabricated using standard ultraviolet (UV) lithography with low cost and easily accessible equipment, which results in them being easy to scale up, and inexpensive to fabricate. Contact-free actuation of freestanding magnetic hollowbots were demonstrated by using an applied 900 G external magnetic field to achieve the movement control in an aqueous environment. According to the movement clip, the average speed of the magnetic hollowbots was estimated to be 1.9 mm/s.

## 1. Introduction

In the past decades, there were normally a couple of routes of the drug delivery system: oral, transdermal, inhalation and injection. Following the rapid development of nanomaterials, micro-nanotechnology, biomedical technology, polymer science, physical chemistry, and so on, a targeted drug delivery system was proposed [1]. 

**Figure 1:**
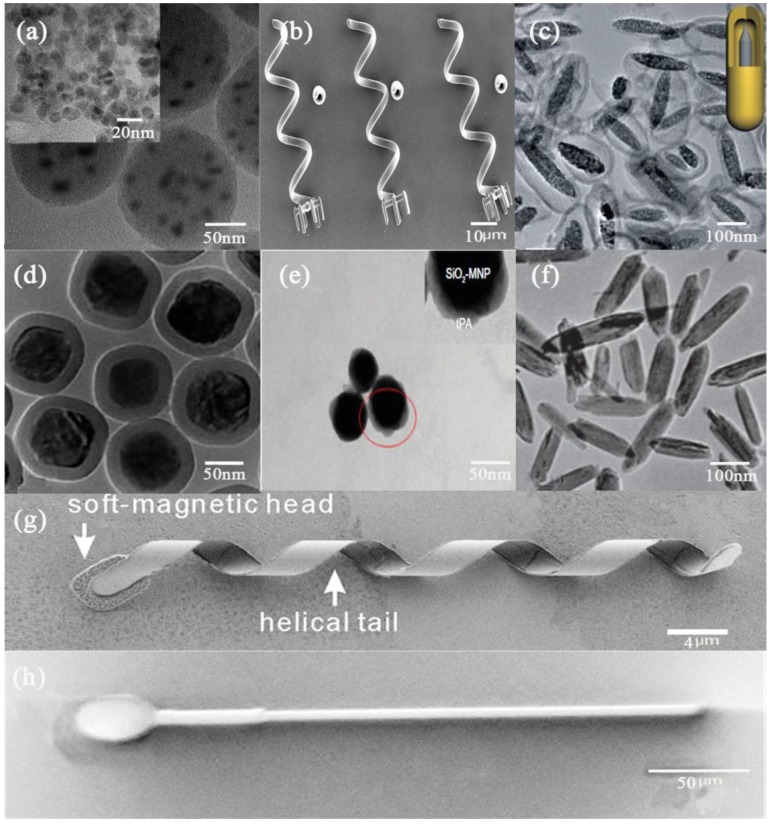
Recent magnetic micro-/nano- drug delivery systems. (**a**) The cyclo(Arg-Gly-Asp-D)-doxorubicin/verapamil-magnetic nanoparticles-Poly(lactic acid-coglycolic acid) nanoparticles. Reproduced with permission from [10]. Copyright 2013, Elsevier; (**b**) An array of helical micromachines. Reproduced with permission from [9]. Copyright 2012, John Wiley and Sons; (**c**) Heterogeneous rattle-type Fe_3_O_4_@mSiO_2_ nanoparticles. Reproduced with permission from [11]. Copyright 2011, The Royal Society of Chemistry; (**d**) Rattle-type magnetic mesoporous silica nanospheres. Reproduced with permission from [12], Copyright 2011, The Royal Society of Chemistry; (**e**) Tissue plasminogen activator bound to silica-coated magnetic nanoparticle after staining with phosphotungstic acid. Reproduced with permission from [13]; (**f**) Hollow core, magnetic, and mesoporous double-shell nanostructures. Reproduced with permission from [14]. Copyright 2011, John Wiley and Sons; (**g**) An untethered artificial bacterial flagella. Reproduced with permission from [15]. Copyright 2009, AIP Publishing.; (**h**) MagnetoSperm moving under the influence of the oscillating weak magnetic fields. Reproduced with permission from [16]. Copyright 2014, AIP Publishing.

A targeted drug delivery system can prolong, localize, target and have a protected drug interaction with the diseased tissue. There are various types of drug delivery vehicles, such as polymeric micelles, liposomes, dendrimers, nanotubes, and microstrucutures, and so on. Due to their small size, high solubility and simple sterilization, polymeric micelles [2] are considered to be an ideal carrier for poorly water soluble drugs. But the physical stability of this carrier is a critical issue of polymeric micelles being microcarriers. Liposomes [3] are one of the most widely studied targeted delivery system, providing advantages such as low immunogenicity, biocompatibility, cell specificity and drug protection. On the other hand, there are also shortcomings such as poor scale-up, cost, short shelf life, and in some cases toxicity and off target effects. Due to their nanoscopic size, narrow polydispersity index, excellent control over molecular structure, availability of multiple functional groups at the periphery and cavities, dendrimers [4] have great potential in delivery carriers. But the high manufacturing cost, safety, toxicity and efficacy issues of dendrimers is still preventing them from clinical applications. Nanoparticles are a great alternative for drug delivery, such as polymer-iron oxide nanocomposites for enhanced permeability and retention (EPR)-independent delivery [5]. Microstructures [6], such as microneedles [7], micropumps [8], microswimmers [9], and so on, have been demonstrated as drug delivery carriers in recent researche; however, these kinds of structures are limited in loaded drug physical state, target location, and loading capability. Some recent magnetic micro-/nano drug delivery systems are summarized in Figure 1.

Magnetic forces offer an attractive option for actuation in microelectromechanical systems (MEMS) and microscale systems because they scale favorably at micro- and nano-scale lengths [17,18]. The contact-free nature of magnetic actuation makes it ideal for applications where contamination must be avoided, such as interactions with cells or other biological samples [19], or where connecting the power source to the actuator would be cumbersome, such as freestanding microrobots [20]. And, unlike systems based on electrostatic or dielectric forces, magnetic actuators can operate in liquid or gas and are unaffected by the ionic concentration of the surrounding medium. 

The benefits of microscale magnetic actuation have led to its implementation in a variety of MEMS and microfluidics devices, performing tasks such as wireless microfluidic mixing [21] and pumping of fluids [22]; in microrobotics, the use of magnetic force to provide wireless control and power is particularly appealing as it does not require the robots to be operated on a specialized surface and it can be used to perform complex three-dimensional motions [23]. Remotely-controlled microscale robots show potential in a variety of applications, including interaction with samples in lab-on-a-chip systems, navigation [16], in vivo delivery of cancer therapies, and performance of retinal- and neuro-surgical procedures [24]. But for many of these applications, ferro- or paramagnetic components are challenging to integrate into existing fabrication schemes.

Researchers have demonstrated various methods for creating magnetic microstructures, most of which can be characterized as either additive, subtractive, or polymer mixing methods. Deposition of magnetic materials via sputtering or evaporation [25] to achieve the microstructure magnetic. These methods could result in good purity of magnetic materials for small samples, but they are expensive and time-consuming for large ones. Etching and cutting [26] are subtractive methods to obtain magnetic microstructures. These methods can achieve good density of magnetic materials, but lack in precision. Polymer mixing [27] is a promising method for magnetic microstructure fabrication, but are expensive for many applications.

Our work builds on the Nd-Fe-B magnetic composite prepared and characterized in previous publications [28]. The paper will show the schematic of the microfabrication method for creating magnetic hollowbots in two geometries, and will also report on characterizations which provide control guidelines for actuating the hollowbots in aqueous environments. The method reported here differs from liposome, nanoparticles, micelle, nanotubes, and so on, used in previous works for loading and delivering drugs; in fabricating hollow microstructures as carriers for drug delivery systems in a specific method of using traditional lithography; and in the structures of the carriers and their materials, geometries, capacity and loading molecule within the hollowbots.

## 2. Materials and Methods

### 2.1. Magnetic Composite Preparation

The magnetic composite materials were made using Nd-Fe-B particles with 2 µm average diameter (Magnequench MQFP-B-20076-084, Molycorp Inc., Greenwood Village, CO, USA) and SU-8 negative photoresist (MicroChem Corp., Westborough, MA, USA) with 4500 mm^2^/s kinematic viscosity. Composites were prepared by mixing 40% concentration Nd-Fe-B powder into SU8 by mass fraction in microcentrifuge tubes. To attain a uniform dispersion and avoid settling of the magnetic particles, samples were mixed by vortexing (Vortex Genie 2, Scientific Industries, Inc., Bohemia, NY, USA) at 3000 rpm for 45 min immediately before use. Unless otherwise stated, particle concentrations are given by fractional mass percentage in the following text.

### 2.2. Hollowbots Fabrication

**Figure 2:**
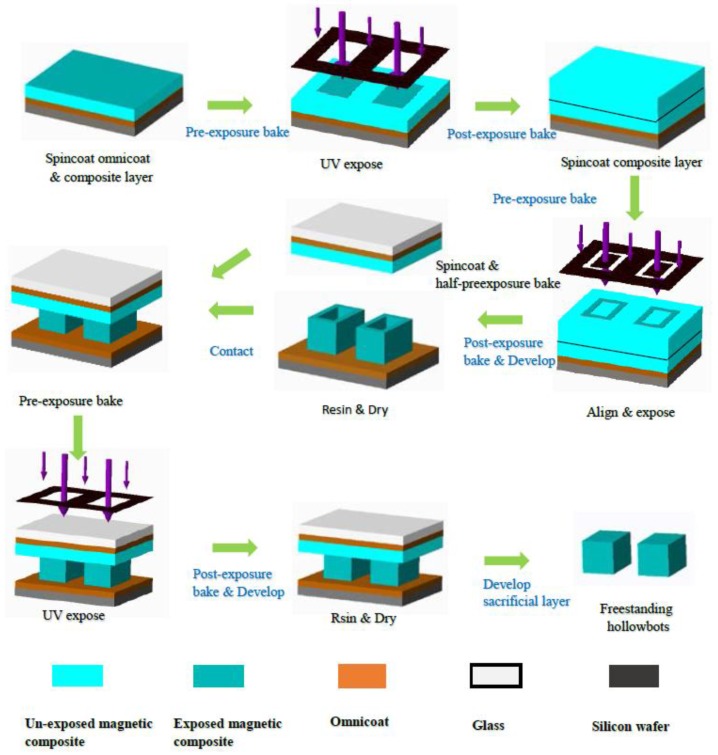
Schematic of magnetic hollowbots fabrication.

The microfabrication process for hollowbots is a facile fabrication process for the construction of fully encapsulated and hollow microstructures using affordable and easy accessible patterned photolithographic processes. The basic idea is to construct a fully encapsulated space inside the microstructures using photopatternable layers and walls by stacking lithography. First, a sacrificial layer was spin-coated with Omnicoat (MicroChem) spin-coating at 500 rpm for 5 s, followed by 3000 rpm for 30 s, and baking at 200 °C for 1 min, in accordance with the manufacturer’s instructions. After the wafer cooled to room temperature, the bottom of the hollowbots was fabricated by spin-coating the mixed magnetic composite on the Omnicoat surface at 500 rpm for 5 s (ramp rate of 100 rpm/s) followed by 30 s at 4000 rpm (ramp rate of 300 rpm/s). This was followed by pre-exposure bake, UV exposure, and post-exposure bake that were all conducted according to the manufacturer’s recommendations for pure SU-8, unless otherwise specified. Then another magnetic composite was applied onto the exposed layer and UV patterned following the same procedure for the first layer but exposed by a hollow rectangle to obtain the side walls of the hollowbots. On the other hand, the lid of the hollowbots was prepared on a cover slip with an Omnicoat sacrificial layer and a half pre-exposure baked magnetic composite layer following the same procedure as previously demonstrated. After development of the two exposed layers in SU-8 developer (MicroChem) with gentle agitation, the half pre-exposure baked lid was flipped and brought it into contact with the base structure, followed with pre-exposure bake, UV exposure through the transparent cover slip, curing the “lid” geometry while maintaining an internal void, and post-exposure bake. Detailed procedure flow is shown in Figure 2.

**Figure 3:**
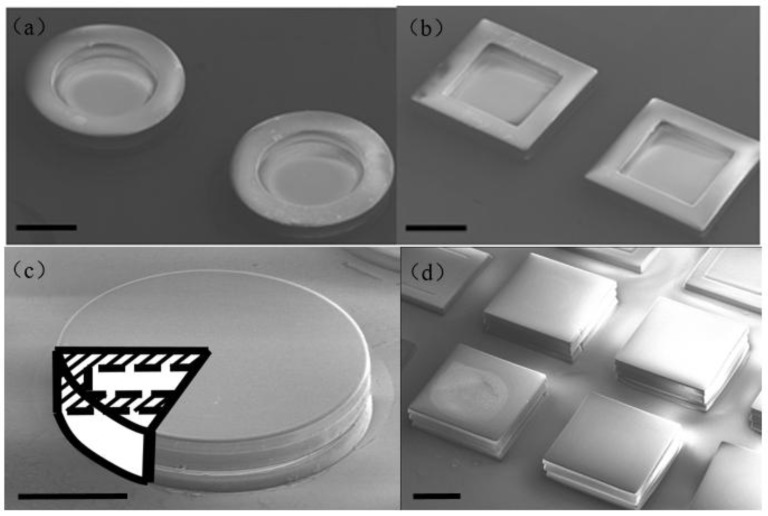
Scanning electron microscope (SEM) images of cylindrical (**a**) and cuboid (**b**) magnetic hollowbots before photopatterning top layer. (**c**) SEM image and sketch of the photopatterned magnetic cylindrical hollowbot structure. (**d**) SEM image of the photopatterned magnetic cuboid hollowbots. Scale bars = 100 µm.

Freestanding magnetic hollowbots were released from the silicon surface and cover slip by chemically dissolving the sacrificial Omnicoat layer by soaking in PG Remover (MicroChem) for up to 45 min under gentle agitation. Individual robots were then carefully separated out using a micromanipulator. Alternatively, the PG Remover solution containing the released microrobots was pipetted into a microcentrifuge tube where a permanent magnet was used to collect the robots to the side of the vial; several wash steps were performed to remove the PG Remover and replace it with isopropyl alcohol for long-term storage. Scanning electron microscope (SEM) images of cylindrical and cuboid magnetic hollowbots are shown in Figure 3.

## 3. Characterization

**Figure 4:**
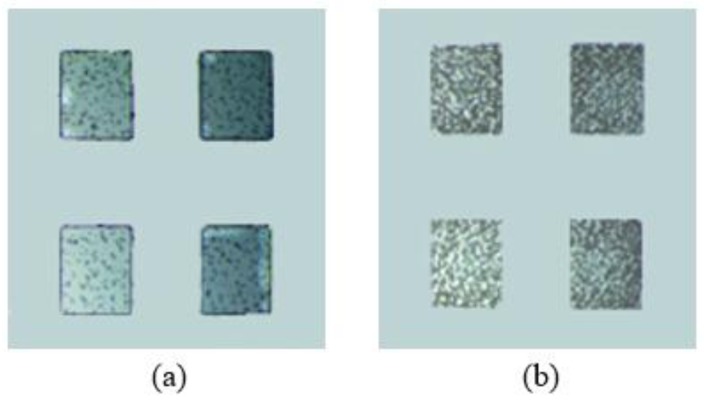
Optical images of (**a**) 40%, 1000 rpm, (**b**) 40%, 4000 rpm samples.

**Figure 5:**
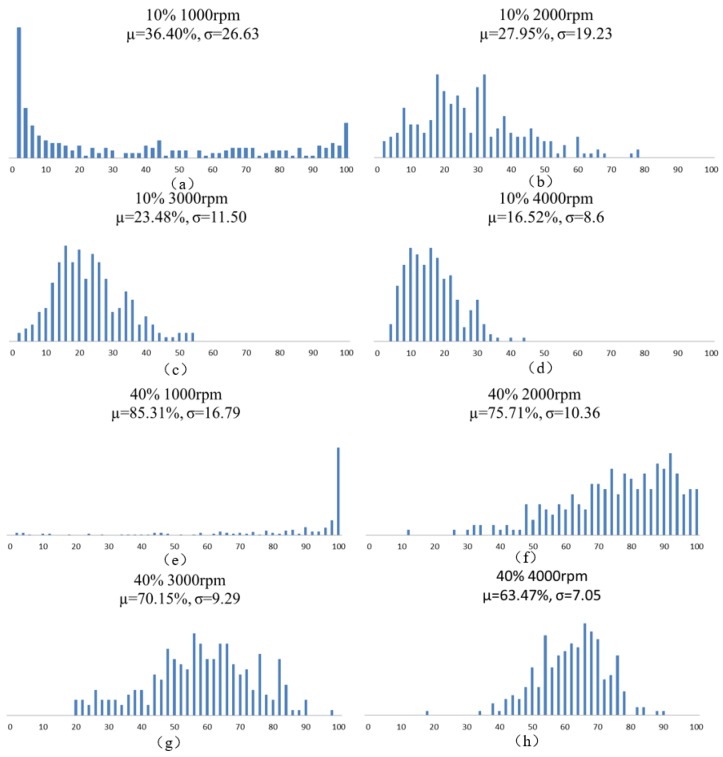
(**a**–**d**) 10% mass fraction Nd-Fe-B composite uniformity for different fabrication spincoating speed. Histograms represent for samples at given processing parameters. (**e**–**h**) 40% mass fraction Nd-Fe-B composite uniformity for different fabrication spincoating speed. Histograms represent for samples at given processing parameters.

To obtain the optimal magnetic actuation performance of these hollowbots, uniform distribution of magnetic particles in the hollowbots is important for obtaining homogeneous magnetic properties, which could be characterized by the method developed in our previous publication [17], analyzing the optical microscopy images of the magnetic composite produced at various particle concentrations and spin speeds. Images taken by Nikon ECLIPSE Ci-POL (Nikon Instruments, Tokyo, Japan), and digitally processed using ImageJ software (1.8.0, National Institutes of Health (NIH), Bethesda, MD, USA) to dice each 250 µm × 250 µm cured magnetic composite area into 10 µm × 10 µm subsets and then calculate the percentage of each subset occupied by magnetic particles (black part of the optical image); and there are 625 subsets. The average µ and standard deviation σ of this value was calculated for set of processing parameters. As particle concentration increases, we would expect µ → 100% and as material uniformity increases, we would expect σ → 0. Optical images for 40% samples with 1000 and 4000 rpm are shown in Figure 4. The statistical parameters, histograms of values for each sample are shown in Figure 5.

The experiment data analysis has shown that the Nd-Fe-B particles are favor to cluster together and create large pieces at lower spincoat speed; while at higher speed, it is much easy to distribute the magnetic composite uniformly. However, it also decreases the microstructure thickness. Magnetic structure fabricated at a higher spin speed will have fewer magnetic particles included; therefore, to create hollowbots for magnetic actuation, each deposited layer would be better to spincoat at high speed with good particle distribution and high particle count, multiple layers of the magnetic composite can be deposited sequentially on a silicon wafer and patterned with UV exposure to achieve the wanted thickness. A microfluidic testing was carried out to confirm the quality of bonding and the leakage of the hollowbots chamber. The test consisted of inserting an ink into the chamber and the hollowbots could withstand pressure up to 400 kPa without any liquid leakage.

## 4. Actuation

**Figure 6:**
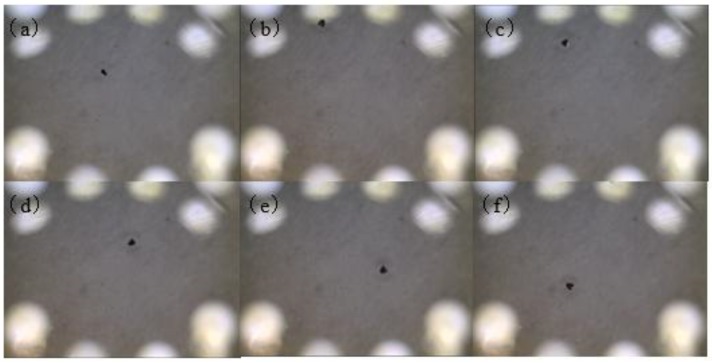
Captured images (every 2 s) from the clip of hollowbots actuated by external magnetic field in isopropyl alcohol. (**a**) 0 s; (**b**) 2 s; (**c**) 4 s; (**d**) 6 s; (**e**) 8 s; (**f**) 10 s.

Freestanding hollowbots were obtained by releasing from the wafer and coverslip as described in previous session and transferred with gentle pipetting to a glass dish filled with isopropyl alcohol. With trapped air in the chamber, hollowbots could float in the isopropyl alcohol environment. An external magnetic field was chosen to actuate the Nd-Fe-B hollowbots, which was generated by a permanent magnet (Nd-Fe-B 56, 12 mm × 12 mm × 12 mm, K&J Magnetics, Inc., Pipersville, PA, USA) with surface field strength of 5600 G held at a distance of 10 mm from the hollowbot. At this distance, the strength of the magnetic field was approximately 900 G, which is able to overcome surface friction, adhesion forces and the drag force applied on the microrobots. The movement of the permanent magnet was held by hand and used to guide the hollowbot move. Using this method, we were able to successfully guide magnetic hollowbots at an average speed of 1.9 mm/s, estimated from the movement clip. The captured images from the magnetic hollowbots actuation clip are shown in Figure 6.

## 5. Conclusions

In this paper, a facile microfabrication of magnetic Nd-Fe-B hollow microrobots was developed by depositing magnetic composite sequentially and patterning the bottom and the side walls of the hollowbots. Contacting with the prepared composite walls on the coverslip and photopatterning the top layer, the magnetic hollowbots are created. In characterization of the uniform distribution with spin speed and particle concentration, it was found that particles could be uniformly dispersed within the polymer matrix by using high spin speed during fabrication without requiring chemical modification of either of the two composite components. The contact-free actuation of the magnetic microrobots was also demonstrated by moving a freestanding hollowbots in an aqueous environment with an external magnetic field. According to the clip, the average speed of the hollowbots could reach 1.9 mm/s. The composite magnetic hollowbots demonstrated here can be applied in a variety of applications where wireless actuation is necessary, including microfluidics, microrobotics, and drug delivery.
